# Treatment of neovascular age-related macular degeneration: insights into drug-switch real-world from the Berlin Macular Registry

**DOI:** 10.1007/s00417-022-05952-8

**Published:** 2023-01-12

**Authors:** Tommes Riemer, Dominique Berndt, Alexander Böker, Josefine Lehmann, Ulrike Schrifl, Saskia Rau, Anne Rübsam, Antonia M. Joussen, Oliver Zeitz

**Affiliations:** Department of Ophthalmology, Charité Universitätsmedizin Berlin, Corporate Member of Freie Universität Berlin, Humboldt-Universität zu Berlin and Berlin Institute of Health, Berlin, Germany

**Keywords:** Aflibercept, Bevacizumab, Ranibizumab, VEGF switch, Vascular endothelial growth factor, Exudative age-related macular degeneration

## Abstract

**Purpose:**

Bevacizumab, ranibizumab, and aflibercept are commonly used to treat neovascular age-related macular degeneration (nAMD). The results of various interventional, mostly randomized head-to-head studies, indicate statistical non-inferiority of these three drugs. The results of these studies are often interpreted as the three drugs being freely interchangeable, resulting in some health systems to pressure ophthalmologists to preferentially use the less expensive bevacizumab. This study analyzes switching from aflibercept or ranibizumab to bevacizumab and back under real-world conditions in order to investigate the assumption of interchangeability of the drugs.

**Methods:**

Treatment data of IVT patients with diagnosed nAMD were extracted from the clinical Berlin Macular Registry database. Patients who underwent a drug switch from aflibercept or ranibizumab to bevacizumab were subject of this study. Statistical comparisons were pre-planned for best corrected visual acuity, central retinal thickness, macular volume, and length of injection interval. Additional endpoints were analyzed descriptively.

**Results:**

Mean visual acuity decreased from 0.57 ± 0.05 under aflibercept/ranibizumab to 0.68 ± 0.06 logMAR after the switch (*P* = 0.001; *N* = 63). CRT increased from 308 ± 11 µm to 336 ± 16 µm (*P* = 0.011; *N* = 63). About half of the subjects were switched back: visual acuity increased from 0.69 ± 0.08 logMAR to 0.58 ± 0.09 logMAR (*N* = 26). CRT decreased from 396 ± 28 to 337 ± 20 µm (*N* = 28).

**Conclusion:**

The data provides real-world evidence that there is loss of visual acuity and an increase in retinal edema after switching to bevacizumab. Thus, the assumption of free interchangeability cannot be confirmed in this cohort.

**Supplementary Information:**

The online version contains supplementary material available at 10.1007/s00417-022-05952-8.


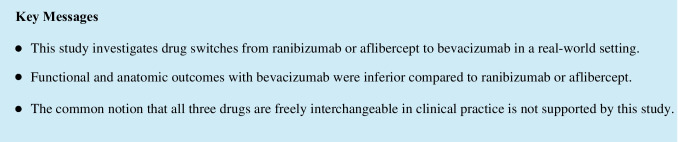


## Introduction

Vascular endothelial growth factor (VEGF) inhibition through intravitreal injections has revolutionized the treatment of neovascular AMD (nAMD). While subjects suffering from nAMD inevitably went blind in the pre-anti-VEGF-era, intravitreal VEGF inhibition not only maintained vision in nAMD, but even improved visual function in a majority of subjects [1–3].

Over the past two decades, four VEGF-inhibitors have been approved for the treatment of neovascular AMD: pegaptanib, ranibizumab, aflibercept, and, as of lately, brolucizumab and faricimab. Of these, Ranibizumab pioneered the field, with proven superiority to the past standard-of-care, i.e., photodynamic therapy(PDT) for predominantly classic choroidal neovascularization (CNV) (ANCHOR study) and observation for occult CNV (MARINA study) [4, 5]. However, to achieve maximum efficacy, ranibizumab was required to be administered monthly, which proves to be challenging, if not infeasible, in clinical routine [6]. Hence, flexible, individualized dosing regimens were established to reduce the number of injections [7, 8]. Parallel to ranibizumab, the cancer drug bevacizumab gained importance as a cost-effective off-label alternative. While a formal pivotal study program for bevacizumab and regulatory approval is lacking, there is broad evidence from multiple studies suggesting non-inferior efficacy compared to ranibizumab [8–11]. Hence, off-label bevacizumab is an integral component of present standard-of-care globally. A few years later, aflibercept was demonstrated to be clinically equivalent efficacy to ranibizumab if administered every second month after an initiation phase of three-monthly injections (VIEW-studies) [12, 13]. The latest addition to the armamentarium of treatment options are brolucizumab and faricimab. The pivotal studies of brolucizumab indicated an option for an early extension of the treatment interval to three months in about half of the patients, while the remaining subset of patients can be treated on a two-month interval [14]. Faricimab was also recently approved and the pivotal program suggests interval extensions out to four months in 45% of subjects [15].

According to these pivotal study programs, the treatment interval is the main differentiator between the approved drugs.

Yet, despite these great advances in therapy, nAMD continues to have its challenges. The invasive administration route through intravitreal (IVT) injections continues to result in a desire to minimize the number treatments. While the pivotal studies mainly employed fixed dosing schemes, real-world treatments are being performed with flexible dosing schemes [16, 17]. If executed rigorously, flexible schemes, particularly treat-and-extend schemes, lead to comparable outcomes to fixed dosing schemes [6, 18, 19]. However, the key risk of any flexible scheme is undertreatment for various reasons such as missed or delayed visits [16, 17]. Therefore, visual function may deteriorate despite IVT treatments.

Tachyphylaxis is another often presumed reason for the deterioration of visual function [20, 21]. Since the available anti-VEGF drugs showed comparable efficacy, they are considered freely interchangeable. Hence, several Ophthalmology societies, such as the German Ophthalmological Society, recommend the switch to another anti-VEGF drug in their guidelines, if an insufficient therapeutic effect is observed despite consistent therapy [22]. This is supported by studies, indicating an improvement in structural outcomes after switch of recalcitrant patients to another anti-VEGF drug [23–28].

The mechanisms of improved efficacy after switching between two anti-VEGF drugs can be explained by different molecule sizes and associated transport through the retina and into the subretinal space (ranibizumab compared with bevacizumab) or different binding characteristics [29].

Thus, the main motivation for a switch is to tackle the unsatisfactory treatment response of recalcitrant patients to ranibizumab or bevacizumab [30–32]. The results of most of these studies showed an anatomical benefit after the switch in terms of central retinal thickness and pigment epithelium detachment characteristics, whereas functional outcomes were variable [30, 31].

The potential interchangeability of the drugs leads to a second motivation to change between anti-VEGF drugs. There is a general debate about whether the higher cost of approved drugs is justified or whether patients should be switched to the less expensive and off-label bevacizumab [33–35]. Drug costs of off-label bevacizumab are by roughly a factor of 20 lower than the costs for the two approved drugs [36]. Hence, drug costs are one of the most dominant topics since the advent of this class of drugs in ophthalmology. It has led to the initiation of large scale randomized clinical trials such as the pioneering CATT and IVAN trials [9–11]. CATT, IVAN, and others suggested equivalent efficacy and safety of bevacizumab compared to ranibizumab.

As this is a highly relevant question from a health-economic standpoint, this point is repeatedly raised by sick funds, physicians and patients [37]. In an increasingly aging society, cost efficiency is of great importance for the stability of the social security system. Thus, these studies influenced political decisions to incentivize or even mandate the use of off-label bevacizumab [38–40]. Several countries, such as France, adjusted their legislation in order to enable and facilitate off-label use of bevacizumab to reduce costs [39]. This occurs under the assumption of clinical equivalence of all available anti-VEGF compounds and in particular the equivalence of off-label bevacizumab. In addition to the influence of the discussion at the political level, this topic, which is discussed in both public and professional circles, also has an influence on drug selection by the treating physician. While there is plenty of data on switches from bevacizumab to ranibizumab or aflibercept, there is a data gap for switches into the other direction. Therefore, this work investigates real-world-experiences with drug switches from ranibizumab or aflibercept to bevacizumab and back.

## Methods

This study was designed as a retrospective, monocentric, real-world study. The source data is archived in the Electronic Medical Record (EMR) system of the Charité Universitätsmedizin Berlin (i.s.h.med, Cerner, München, Germany). Imaging data is stored in the Heidelberg Eye Explorer (Heidelberg Engineering, Heidelberg, Germany). For the present study, data was extracted into a separate clinical study database, in the following referred to as Berlin Macula Registry. The primary data capture was done in the REDCap electronic Case Report From (eCRF) system. For analysis, data was extracted from REDCap and transferred into appropriate statistical analysis software. All data handling was done within the safeguarded Charité IT environment to comply with data protection law. The study was approved by the ethics committee of Charité (reference number: EA1/085/20) and has been reviewed by the data protection committee of Charité. Consent for use of data was obtained from each patient.

### Eligibility

All patients, who underwent treatment for nAMD between 01-JUL-2017 and 31-JAN-2020, were reviewed. Subjects, who underwent a switch from aflibercept or ranibizumab to bevacizumab between 01-JUL-2017 and 31-JAN-2020, were included into the analysis set for this study. Detailed inclusion criteria were as follows: patient age > 50 years, drug switch from either aflibercept or ranibizumab to bevacizumab, at least three injections with the same drug before and after switch, interval between last injection of aflibercept or ranibizumab, and first injection of bevacizumab < 5 months. Exclusion criteria involved any type of ocular surgery between the first out of three IVT injections before switch and last out of three IVT injections after switch as well as additional presence of long-term anti-VEGF-indicating disease in the study eye other than nAMD. If both eyes of one patient were eligible, the eye receiving more IVT injections in total was selected.

### IVT injections

All IVT injections took place at one of the Charité IVT injection centers. The treatment algorithm and the IVT injection procedure itself were done in accordance with recommendations of the German Ophthalmological Society [22]. In brief, it includes an upload phase of three injections at a 4-week interval followed by maintenance therapy using the Treat and Extend algorithm [22]. In the latter, the treatment interval is adjusted stepwise by 2-week increments/decrements depending on disease activity. If no disease activity is seen at an injection interval of 12 weeks or longer, cessation of therapy is considered.

### Drugs and choice of drug

The choice of the drug was at the discretion of the treating physician after considering patient preferences as well as reimbursement conditions of the patient’s health insurance. Commercially available presentations used for therapy were ranibizumab (Lucentis®, Novartis Pharma AG, Basel, Switzerland) and aflibercept (Eylea®, Bayer Pharma AG, Berlin, Germany). Off-label Bevacizumab (Avastin®, F. Hoffmann-La Roche AG, Basel Switzerland) was prepared for intravitreal use by the pharmacy of Charité under sterile conditions.

### Objectives

The objective of the study was to investigate the impact of the switch on functional outcomes (primary objective), anatomic outcomes, and treatment interval.

### Endpoints

Visual acuity and OCT measurements were recorded for each visit as available. Visit and injection dates were collected and analyzed to describe the treatment interval.

The main analysis was focused on functional and morphological outcomes immediately before and after the switch from aflibercept or ranibizumab to bevacizumab. The primary endpoint was the change in visual acuity (logMAR). Secondary endpoints were the change in central retinal thickness (central ETDRS subfield), the change in macular volume, as well as the treatment interval. The treatment interval was defined as the last observed treatment interval on the respective drug. This definition was chosen in order to capture a treatment interval under steady-state conditions.

Furthermore, a qualitative fluid compartment analysis of all OCT images was performed [41]. Criteria for the compartment analysis are shown in Table S1 (Supplemental Digital Content 1). The results of the compartment analysis were summarized descriptively.

To determine robustness of the results, several sensitivity analyses for all endpoints were performed. The definitions of these sensitivity analyses are reported along with their results in the supplemental material.

### Second switch

Some subjects experienced a second switch, i.e., a switch from bevacizumab back to aflibercept or ranibizumab. This second switch was analyzed descriptively using the same metrics and systematics as for the first switch. An informal comparison with the outcomes in those subjects staying on bevacizumab for the entire observation period was performed.

### Statistical analysis

Unless stated otherwise, data is displayed as mean ± standard error of means. To analyze switch results, statistical tests on the following four variables were pre-planned and performed (before first switch vs. after first switch): (1) best corrected visual acuity, (2) central macular thickness, (3) macular volume, and (4) treatment interval. These four Wilcoxon tests were done only for the primary switch from ranibizumab or aflibercept to bevacizumab. The significance level (alpha) was chosen to be 0.05. To correct for multiplicity, Bonferroni adjustment was done. Consequently, *P* < 0.0125 was regarded to be statistically significant. As this is an exploratory real-world-study, no formal sample size calculation was done. A 95% confidence interval (CI) of the mean is reported for the four variables. All further analyses provided are purely descriptive.

Data was stored in REDCap (Vanderbilt, Nashville, USA). Statistical tables were created by using SQL-functions of Microsoft Access (Version 2008; Microsoft, Seattle, USA). Statistical analysis was performed with SPSS Version 25 (IBM, Armonk, USA). Descriptive statistics were performed with Microsoft Excel Version 16.45 (Microsoft, Seattle, USA). Figures were produced using PrismGraph (GraphPad, San Diego, USA).

## Results

**Fig. 1:**
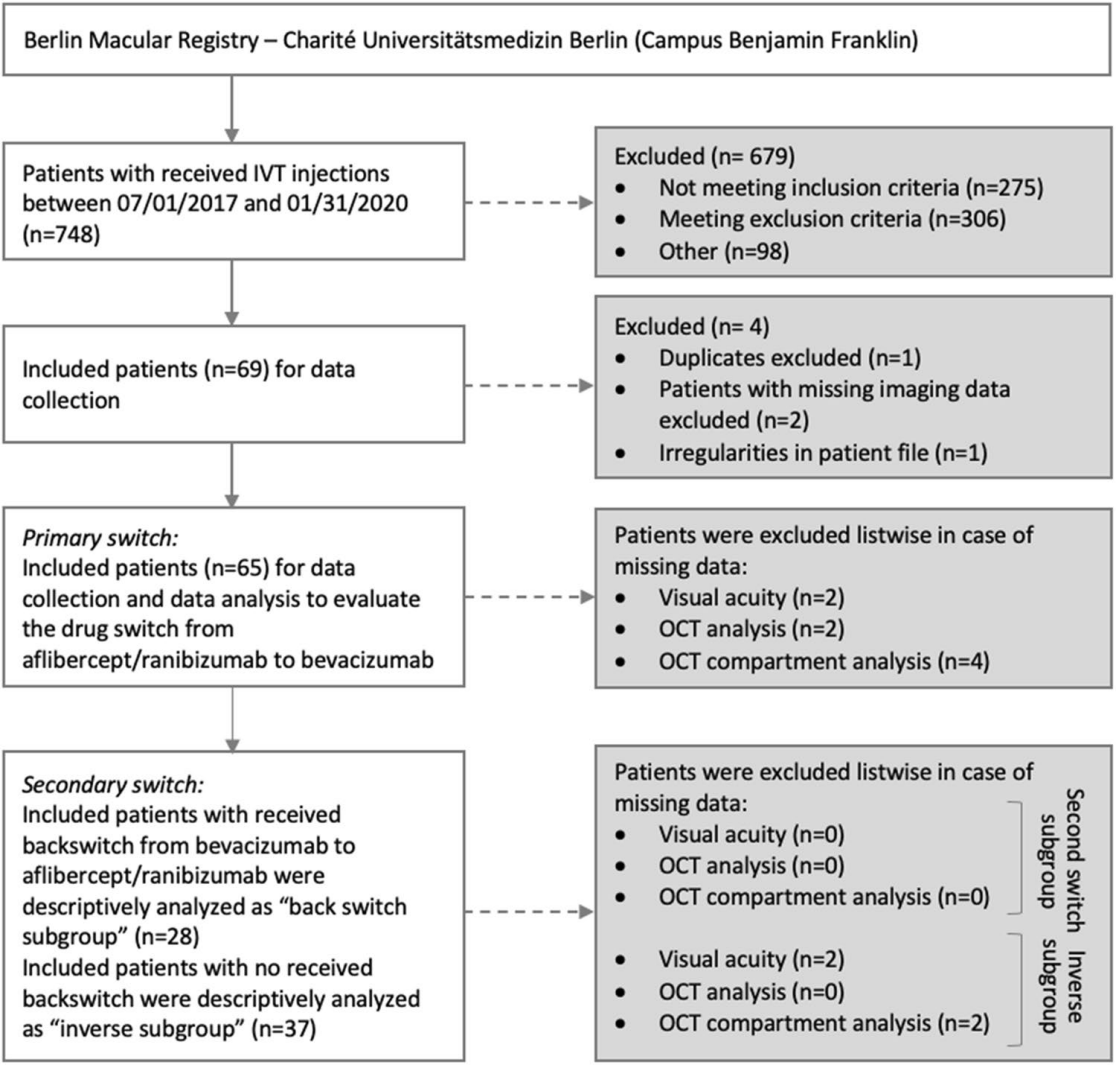
Recruitment process

A total of 748 patient files has been reviewed, 69 of which qualified for this analysis. Two patients were excluded, because of insufficiently available follow-up data. One dataset turned out to be a duplicate and was excluded. One further patient was excluded due to a lack of imaging in the patient´s file. This results in a final number of 65 subjects being eligible for the analysis (CONSORT chart in Fig. 1).

The mean age of the eligible subjects was 80.8 ± 1.1 years. 61.5% (*n* = 40) of the patients were female and 38.5% (*n* = 25) male. There were 29 (44.6%) right study eyes and 36 (55.4%) left study eyes included. Demographic and clinical characteristics of the patients are described in Table 1. Concomitant diagnoses and surgical history on the study eye are shown in supplemental material (Table S2; Supplemental Digital Content 2).

**Table 1:** Demographic and clinical characteristics of study population

		Total group (*N* = 65)
Sex	Male	25 (40%)
	Female	40 (60%)
Age [in years]^a^	Mean	78.9 ± 1.1
	Median	77.8 (62.1; 98.1)
Study eye	Right Eye	29 (44.6%)
	Left Eye	36 (55.4%)
Visual acuity^a^ [in LogMAR]	Mean	0.57 ± 0,05
	Median	0.4 (0;1,5)
Intraocular pressure^a^ [in mmHg]	Mean	13.9 ± 0.3
	Median	14 (9;20)
CRT [in µm]^a^	Mean	310.1 ± 11.0
	Median	293 (129;619)
Macular volume^a^ [in mm^3^]	Mean	8.1 ± 0.14
	Median	8.1(5.2;11.4)
Treatment time [in years]	Mean	4.8 ± 0.3
	Median	4.6 (0.6;9.8)
IVT injections	Mean	29.5 ± 1.7
	Median	28 (6;64)

^a^Baseline = date of the last IVT injection before switch to bevacizumab

**Fig. 2:**
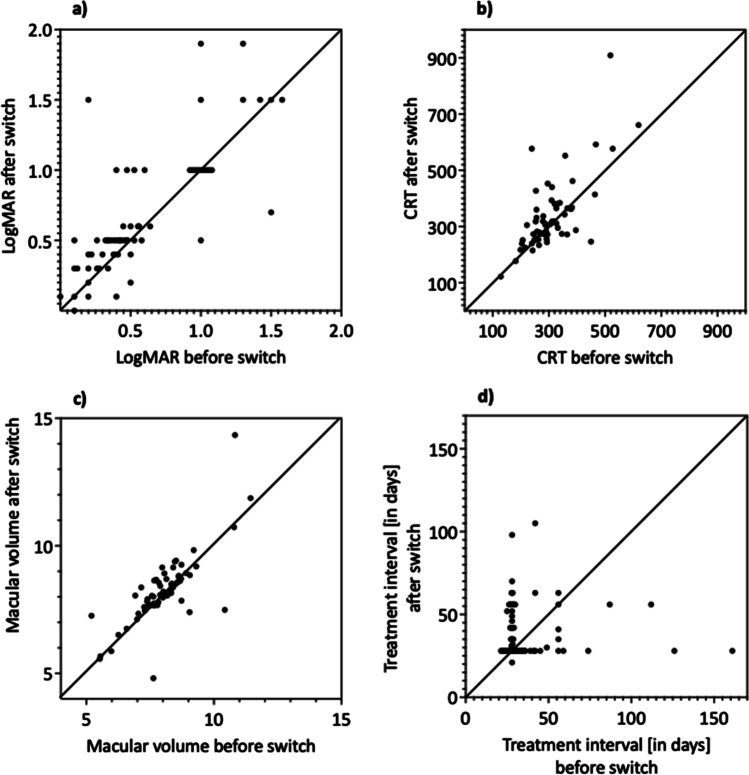
Results of primary switch

**Fig. 3:**
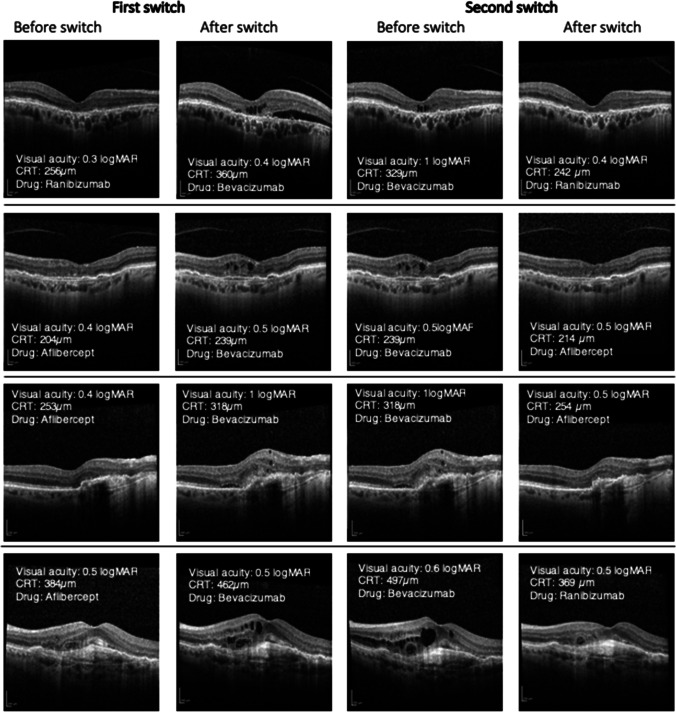
Representative OCT images

When switched from aflibercept or ranibizumab to bevacizumab, best-corrected visual acuity decreased significantly from 0.57 ± 0.05 (20/74.2; CI [0.48, 0.68]) to 0.68 ± 0.06 (20/95.7; CI [0.57, 0.79]) logMAR (*P* = 0.001; *N* = 63). Two patients had to be excluded due to missing data. This vision decrease was accompanied by morphological deterioration. CRT increased significantly from 307.59 ± 10.7 (CI [286.18, 329]) µm to 335.95 ± 15.84 (CI [304.3, 367.61]) µm (*P* = 0.011; *N* = 63) and the macular volume from 8.03 ± 0.14 (CI [7.74, 8.32]) mm^3^ to 8.24 ± 0.14 (CI [7.9, 8.58]) mm^3^ (*P* < 0.001; *N* = 63). Two patients had to be excluded due to missing data. The treatment interval was shortened close to the minimum of 4 weeks (39 ± 3.1 days to 32.8 ± 2.3 days) and, initially, was then gradually extended and reached 39 days 39 ± 3.1 (CI [32.9, 45.1]) days before and 39.8 ± 2.1 (CI [35.6, 44]) days after the switch (*P* = 0.323; *N* = 65). These results are shown in Fig. 2. The OCT-compartment analysis confirmed the trend toward anatomic worsening after the switch to bevacizumab (Table 2; representative OCT images in Fig. 3). The results of the sensitivity analyses are in line with the main analyses (Table S3; Supplemental Digital Content 3).

**Table 2:** Qualitative OCT changes

	OCT criteria	Available before switch	Available after switch	Difference
Primary switch				
Total group - *N* = 61	Foveal depression	51 (83.6%)	48 (78.7%)	-4.92%
	Macular edema	34 (55.7%)	49 (80.3%)	+ 24.6%
	Intraretinal edema	19 (31.2%)	33 (54.1%)	+ 22.9%
	Subretinal edema	18 (29.5%)	29 (47.5%)	+ 18.0%
	RPE^a^ detachment	14 (23.0%)	15 (24.6%)	+ 1.6%
Secondary switch				
Back switch subgroup - First switch - *N* = 28	Foveal depression	23 (82.1%)	22 (78.6%)	-3.5%
	Macular edema	19 (67.9%)	25 (89.3%)	+ 21.4%
	Intraretinal edema	8 (28.6%)	16 (57.1%)	+ 28.5%
	Subretinal edema	12(42.9%)	15 (53.6%)	+ 10.7%
	RPE^a^detachment	5 (17.9%)	6 (21.4%)	+ 3.5%
Back switch subgroup - Second switch - *N* = 26	Foveal depression	21 (80.8%)	23 (88.5%)	+ 7.7%
	Macular edema	25 (96.2%)	18 (69.2%)	-27.0%
	Intraretinal edema	16 (61.5%)	11 (42.3%)	-19.2%
	Subretinal edema	20 (76.9%)	8 (30.8%)	-46.1%
	RPE^a^ detachment	8 (30.8%)	4 (15.4%)	-15.4%
Inverse subgroup - First switch - *N* = 33	Foveal depression	28 (84.9%)	26 (78.8%)	+ 6.1%
	Macular edema	15 (45.5%)	24 (72.7%)	+ 27.2%
	Intraretinal edema	11 (33.3%)	17 (51.2%)	+ 17.9%
	Subretinal edema	6 (18.2%)	14 (42.4%)	+ 24.2%
	RPE^a^ detachment	9 (27.3%)	9 (27.3%)	± 0.0%

^a^RPE, retinal pigment epithelial

### Subgroup analyses: second switch vs. no second switch

Close to half of the subjects (43.1%; *N* = 28) were switched back from bevacizumab to aflibercept or ranibizumab. The mean age of this group (77.6 ± 1.4 years) and gender ratio (F/M = 16/12) was similar to the overall group and the inverse sub-group, i.e., those not undergoing a second switch. Detailed demographics and disposition of the two subgroups are shown in Table S4 (Supplemental Digital Content 4).

As in the overall population, best-corrected visual acuity dropped in this subgroup during the first switch (0.47 ± 0.06 (20/95) vs. 0.6 ± 0.07 logMAR (20/79)). The CRT and macular volume deteriorated (329.4 ± 18.7 vs. 401.0 ± 29.3 µm and 8.2 ± 0.2 vs. 8.7 ± 0.3 mm^3^, resp.). The IVT injection interval remained constant (33.9 ± 2.2 vs. 33.9 ± 2.0 days).

This worsening in three out of four key metrics used in this study was reversible, when treatment with bevacizumab was replaced by treatment with ranibizumab or aflibercept. BCVA increased from 0.69 ± 0.08 (20/98) to 0.58 ± 0.09 logMAR (20/76) (*N* = 26). At the same time, there was a decrease in CRT from 396.3 ± 28.3 to 337.3 ± 19.8 µm and the macular volume from 8.7 ± 0.3 to 8.1 ± 0.2 mm^3^ (*N* = 28). The IVT injection interval increased from 33.9 ± 2.0 to 49.5 ± 6.0 days (*N* = 28). The trend toward improvement was confirmed by OCT compartment analyses (Table 2 and Fig. 3).

In the inverse sub-group, i.e., those subjects staying on bevacizumab for the entire follow-up period, best-corrected visual acuity and OCT compartment analyses showed deterioration at a similar magnitude than in the overall population subsequent to the switch from aflibercept or ranibizumab to bevacizumab. At the same time, CRT and macular volume decreased (Tables 2 and 3). Interestingly and despite functional worsening, the treatment interval was not shortened and in fact on average even slightly extended (42.8 ± 5.1 to 44.2 ± 3.3 days) in this group.

**Table 3:** BCVA and quantitative OC`T-findings in inverse subgroup

Inverse subgroup (*N* = 37)	Before switch	After switch
Visual acuity [in LogMAR]	0.61 ± 0.07	0.76 ± 0.09
CRT [in µm]	290.1 ± 12.1	283.9 ± 11.0
Macular volume [in mm^3^]	7.9 ± 0.18	7.86 ± 0.2

Additional sensitivity analyses on the subgroups (Tables S5 and S6) including analysis by primary drug (Table S7) are summarized in the supplementary material. The results of these sensitivity analyses corroborate the findings of the main analyses.

## Discussion

This real-world study found–consistent through all analyses–a worsening of functional and morphological outcomes in subjects switched from aflibercept or ranibizumab to bevacizumab. In a sub-set of subjects experiencing a switch back from bevacizumab to aflibercept or ranibizumab, the trend towards worsening of functional and morphological outcomes could be reversed.

Switching drugs in IVT treatment of neovascular AMD is an highly relevant topic gaining a lot of attention. Several interventional and real-world studies have been published on this matter. Most studies deal with switches from ranibizumab or bevacizumab to aflibercept and originate from the time, when aflibercept was introduced to the market as second widely used approved VEGF-inhibitor for nAMD.

The main motivation for switching in these studies was unsatisfactory response to treatment with ranibizumab or bevacizumab in recalcitrant patients [30–32].

A second motivation for switching may be cost-saving reasons, which are of such relevance that different studies have already influenced policy decisions promoting or even mandating the use of bevacizumab as off-label [38–40].

In contrast to the quite broad availability of comparative data from RCTs for bevacizumab vs. ranibizumab, only limited real-world data on switches from aflibercept or ranibizumab to bevacizumab is available. A retrospective study by Pinheiro-Costa et al. in which a switch from ranibizumab to bevacizumab was performed in 110 patients with nAMD, and which was performed due to an institutional decision based on economic reasoning, showed a significant decrease in visual acuity as well as a trend towards increase of CRT due to the switch [42]. The reason for the CRT increase is mainly caused by intraretinal fluid and subretinal fluid, which is in line with our results [42].

Two other smaller retrospective studies analyzing the switch from ranibizumab to bevacizumab were largely in line with our results.

Andreoli et al. did not show a significant change but a trend towards better visual acuity and IVT injection intervals under treatment with ranibizumab compared to bevacizumab [43].

In a case series by Yamada et al., 7 patients were switched after three monthly ranibizumab injections to six weekly bevacizumab injections. This study found a non-significant decrease in visual acuity after 6 weeks but a significant reduction in foveal retinal thickness (FRT) after 6 months of therapy with bevacizumab [44].

Comparability of the afore mentioned studies to our study is reduced because the switch from aflibercept to bevacizumab was not included in their studied cohort.

The present study from the Berlin Macula Registry aims to close this gap. In the health-economic environment in Germany, there is occasionally a gentle push towards yet no enforcement of off-label prescriptions of bevacizumab, which also have influenced drug choice for the subjects in this study. While physicians are encouraged to consider health-economic aspects, they still remain free in their ultimate decision. In our study, population consistent deterioration of functional and morphological outcomes was observed. This suggests that RCT results implying clinical equivalence of bevacizumab do not translate into real-world practice outcomes. This finding is in line with limited previous study data from different geographies. Different to previous studies, the present study was not limited to ranibizumab as primary drug and also included aflibercept. This is a relevant differentiator to previous work, as aflibercept is an important pillar of the present standard-of-care.

Besides the main objective of analyzing the outcomes of a switch to bevacizumab, the study disclosed additional interesting aspects of the treatment reality of neovascular AMD subjects. Looking at the sub-group that was not switched back to aflibercept or ranibizumab, it is striking that the functional and anatomic outcomes are as such that the response to the treatment with bevacizumab must be regarded as unsatisfactorily. In hindsight, one would have expected that also this population would have been switched back or that the treatment interval would have at least been shortened. In some cases, this may be due to development of atrophy as indicated by reduction of mean CRT. However, others may have benefitted from a switch-back. This illustrates the main risk of any flexible treatment regimens: Subtle signs of worsening may be overlooked leading to under-treatment.

Of course, the present study also has its limitations, mainly those inherent to secondary data collection of real-world data. The treatment did not follow a prospectively developed protocol, which, however, is the nature of patient care in a real-world setting. The monocentric approach ensures homogeneity of the data and reduces confounders through different clinical practice across centers. The lack of a control group is mitigated by the analyses of second switch back to aflibercept or ranibizumab. With all caution as the number of cases in the switch-back sub-group is limited, these analyses demonstrate that deterioration of functional and anatomic outcomes is reversible. This observation fits well into the overall picture generated from the totality of data of this study. It corroborates the primary result and strongly suggests that the observed effects be truly attributable to bevacizumab.

The additional sensitivity analyses constitute a further measure to mitigate the shortcomings of a secondary data collection. The combination of data quality and quantity of data points per subject and the large number of additional sensitivity and sub-group analyses allow a robust conclusion on the study cohort. This is supported by the fact that all primary analyses, sensitivity analyses and sub-group analyses show a consistent picture so that the observed effects can be regarded as robust and assumed to be real.

## Summary

This study shows that bevacizumab leads to outcomes inferior to those with aflibercept or ranibizumab under real-world-conditions. It shows that conclusions from RCTs suggesting that drugs are interchangeable do not translate into clinical practice. Given that economics are the sole driving force behind the usage of bevacizumab, this is an important information. This may also be of relevance once decisions on real-world equivalence of biosimiliars have to be made. The observation of this study should be considered by reimbursement decision makers.

## Supplementary Information

Below is the link to the electronic supplementary material.

Supplementary file1 (PDF 53.5 KB)

Supplementary file2 (PDF 66.9 KB)

Supplementary file3 (PDF 66.7 KB)

Supplementary file4 (PDF 102 KB)

Supplementary file5 (PDF 69.7 KB)

Supplementary file6 (PDF 53.1 KB)

Supplementary file7 (PDF 62.2 KB)

## Abbreviations

IVT: Intravitreal
nAMD: Neovascular age-related macular degeneration
CRT: Central retinal thickness
SEM: Standard error of mean
VEGF: Vascular endothelial growth factor
PDT: Photodynamic therapy
CNV: Choroidal neovascularization
EMR: Electronic medical record
eCRF: Electronic case report from
CI: Confidence interval
