# Correction to: Treatment of neovascular age-related macular degeneration: insights into drug-switch real-world from the Berlin Macular Registry

**DOI:** 10.1007/s00417-023-06011-6

**Published:** 2023-02-16

**Authors:** Tommes Riemer, Dominique Berndt, Alexander Böker, Josefine Lehmann, Ulrike Schrifl, Saskia Rau, Anne Rübsam, Antonia M. Joussen, Oliver Zeitz

**Affiliations:** Department of Ophthalmology, Charité Universitätsmedizin Berlin, Corporate Member of Freie Universität Berlin, Humboldt-Universität zu Berlin and Berlin Institute of Health, Berlin, Germany

**Correction to: Graefe’s Archive for Clinical and Experimental Ophthalmology**

10.1007/s00417-022-05952-8 In the published version of this article, two author names contained some errors.

Anne Lehmann should be named “**Anne Rübsam**” and Josefine Urban should be named “**Josefine Lehmann**”.

This is being corrected in this publication.

