# Supplementary material for: Treatment of neovascular age-related macular degeneration: insights into drug-switch real-world from the Berlin Macular Registry
Source: Graefes Arch Clin Exp Ophthalmol. 2023 Jan 12;261(6):1681–90. doi: 10.1007/s00417-022-05952-8 (PMC10198863; doi:10.1007/s00417-022-05952-8)
Supplement: Supplementary file 1 — Supplementary file1 (PDF 53.5 KB) [file 417_2022_5952_MOESM1_ESM.pdf]

Table S1

Criteria for qualitative OCT analysis

Criteria	Assessment
**Image quality**	Optimal/ sufficient/ insufficient
**Foveal depression**	Present/ not present
**Macular edema**	
**Intraretinal fluid**	
**Subretinal fluid**	
**RPE[a] detachment**	

[a]RPE, retinal pigment epithelia
