# Supplementary material for: Treatment of neovascular age-related macular degeneration: insights into drug-switch real-world from the Berlin Macular Registry
Source: Graefes Arch Clin Exp Ophthalmol. 2023 Jan 12;261(6):1681–90. doi: 10.1007/s00417-022-05952-8 (PMC10198863; doi:10.1007/s00417-022-05952-8)
Supplement: Supplementary file 2 — Supplementary file2 (PDF 66.9 KB) [file 417_2022_5952_MOESM2_ESM.pdf]

Table S2

Breakdown of patients with secondary diagnoses (n=60; 90.8%) and operations on the study eye (n=46; 70.8%)

**Secondary diagnoses (N=60)**	**N (%)**
**Cataract**	14 (21,5%)
**Pseudophakia**	41 (63,1%)
**Glaucoma**	14 (21,5%)
**Primary open-angle glaucoma**	4 (6,2%)
**Other glaucoma**	10 (15,4%)
**Retinal artery occlusion**	1 (1,5%)
**Corneal diseases**	1 (1,5%)
**Uveitis**	2 (3,1%)
**Eyelid disease**	5 (7,7%)
**Eye surgery (N=57)**	**N (%)**
**Cataract surgery**	41 (71,9%)
**Laser surgery (ALC[a], YAGb laser iridotomy, YAG laser capsulotomy)**	8 (14%)
**Minimally invasive glaucoma surgery (iStent, trabectome, canaloplasty ab interno)**	1 (1,8%)
**Vitrectomy**	2 (3,5%)
**Eyelid surgery**	2 (3,5%)

[a]ALC, argon-laser-coagulation; [b]YAG, yttrium-aluminum-garnet
