# Supplementary material for: Treatment of neovascular age-related macular degeneration: insights into drug-switch real-world from the Berlin Macular Registry
Source: Graefes Arch Clin Exp Ophthalmol. 2023 Jan 12;261(6):1681–90. doi: 10.1007/s00417-022-05952-8 (PMC10198863; doi:10.1007/s00417-022-05952-8)
Supplement: Supplementary file 3 — Supplementary file3 (PDF 66.7 KB) [file 417_2022_5952_MOESM3_ESM.pdf]

Table S3

Sensitivity analyses for primary switch (n=65):

Switch distance		
**Distance between last A[a]/R[b] and first B[c] [in days]**	80.2± 4.1	

Drug exposure	Before switch	After switch
**Therapy time [in years]**	2.9± 0.3	0.84± 0.1
**Number of IVT-injections**	16.3± 1.5	7.78± 0.5
**Interval between last two recorded IVT-injections [in days]**	39± 3.1	39.8± 2.1

Assigned finding distances	Before switch	After switch
**IVT-injection to BCVA distance [in days]**	37.2± 3.3	37.2± 2.2
**IVT-injection to OCT distance [in days]**	36.8± 2.1	36.2± 1.9

[a]A, aflibercept; [b]R, ranibizumab; [c]B, bevacizumab
