# Supplementary material for: Treatment of neovascular age-related macular degeneration: insights into drug-switch real-world from the Berlin Macular Registry
Source: Graefes Arch Clin Exp Ophthalmol. 2023 Jan 12;261(6):1681–90. doi: 10.1007/s00417-022-05952-8 (PMC10198863; doi:10.1007/s00417-022-05952-8)
Supplement: Supplementary file 4 — Supplementary file4 (PDF 102 KB) [file 417_2022_5952_MOESM4_ESM.pdf]

**Table S4**

Demographic and clinical characteristics of subgroups

| | | Backswitch group (N=28) | Inverse group (N=37) |
|---|---|---|---|
| **Sex** | **Male** | 12 (42.9%) | 14 (37.8%) |
| | **Female** | 16 (57.1%) | 23 (62.2%) |
| **Age [in years]**[a] | **Mean** | 77.6±1.4 | 79.5±1.6 |
| | **Median** | 76.9 (62.1;93.2) | 79.7 (63.5;98.1) |
| **Study eye** | **Right Eye** | 13 (46.4%) | 16 (43.2%) |
| | **Left Eye** | 15 (53.6%) | 21 (56.8%) |
| **Visual aquity**[a] **[in LogMAR\|** | **Mean** | 0.47±0.06 | 0.61±0.07 |
| | **Median** | 0.4 (0;1.5) | 0.5 (0.1±1.5) |
| **Intraocular pressure**[a] **[in mmHg]** | **Mean** | 13.7±0.5 | 13.9±0.4 |
| | **Median** | 14 (9;20) | 13.5 (9;18) |
| **CRT [in µm]**[a] | **Mean** | 329.4±18.7 | 290.1±12.1 |
| | **Median** | 301 (204;619) | 290 (129;464) |
| **Macular volume**[a] **[in mm$^3$]** | **Mean** | 8.2±0.24 | 7.9±0.18 |
| | **Median** | 8.17 (5.6;11.4) | 7.9 (5.2;9.3) |
| **Treatment time [in years]** | **Mean** | 5.5±0.5 | 4.2±0.4 |
| | **Median** | 5.9 (1.2;9.8) | 4.1 (0.6;8.0) |
| **IVT injections** | **Mean** | 34.6±2.6 | 25.5±2.1 |
| | **Median** | 33.5 (10;58) | 24 (6;64) |

[a]Baseline = date of the last IVT injection before switch to bevacizumab
