# Supplementary material for: Treatment of neovascular age-related macular degeneration: insights into drug-switch real-world from the Berlin Macular Registry
Source: Graefes Arch Clin Exp Ophthalmol. 2023 Jan 12;261(6):1681–90. doi: 10.1007/s00417-022-05952-8 (PMC10198863; doi:10.1007/s00417-022-05952-8)
Supplement: Supplementary file 5 — Supplementary file5 (PDF 69.7 KB) [file 417_2022_5952_MOESM5_ESM.pdf]

Table S5

Sensitivity analyses for secondary switch (backswitch group, n=28)

**Switch distance**				
**Primary-switch-distance between last A[a]/R[b] and first B[c] [in days]**	81.1± 5.8			
**Secondary-switch-distance between last A[a]/R[b] and first B[c] [in days]**	52.0± 3.5			

**Drug exposure**	**before switch**	**after switch**	**before switchback**	**after switchback**
**Therapy time [in years]**	3.1± 0.5	0.5± 0.07	0.5± 0.07	0.8± 0.09
**Number of IVT-injections**	17.7± 2.5	5.6± 0.5	5.6± 0.5	7.9± 0.8
**Interval between last two recorded IVT-injections [in days]**	33.9± 2.2	33.9± 2.0	33.9± 2.0	49.5± 6.0

**Assigned finding distances**	**before switch**	**after switch**	**before switchback**	**after switchback**
**IVT-injection to BCVA distance [in days]**	36.1± 3.2	34.9± 2.5	32.4± 1.1	39.2± 3.1
**IVT-injection to OCT distance [in days]**	34.9± 3.0	34.9± 2.5	32.1± 1.0	37.7± 2.6

[a]A, aflibercept; [b]R, ranibizumab; [c]B, bevacizumab
