# Supplementary material for: Treatment of neovascular age-related macular degeneration: insights into drug-switch real-world from the Berlin Macular Registry
Source: Graefes Arch Clin Exp Ophthalmol. 2023 Jan 12;261(6):1681–90. doi: 10.1007/s00417-022-05952-8 (PMC10198863; doi:10.1007/s00417-022-05952-8)
Supplement: Supplementary file 6 — Supplementary file6 (PDF 53.1 KB) [file 417_2022_5952_MOESM6_ESM.pdf]

Table S6

Sensitivity analyses for secondary switch (inverse group, n=37)

**Switch distance**		
**Distance between last A[a]/R[b] and first B[c] [in days]**	79.5± 5.8	

**Drug exposure**	**Before switch**	**After switch**
**Therapy time [in years]**	3.2± 0.4	1.0± 0.1
**Number of IVT-injections**	16.5± 2.0	8.7± 0.7
**Interval between the last two recorded IVT-injections [in days]**	42.8± 5.1	44.2± 3.3

**Assigned finding distances**	**Before switch**	**After switch**
**IVT-injection to BCVA distance [in days]**	38.0± 2.9	39.1± 3.4
**IVT-injection to OCT distance [in days]**	38.4± 2.8	37.1± 2.8

A[a] = aflibercept; B[b] = ranibizumab; C[c] = bevacizumab
