# Supplementary material for: Treatment of neovascular age-related macular degeneration: insights into drug-switch real-world from the Berlin Macular Registry
Source: Graefes Arch Clin Exp Ophthalmol. 2023 Jan 12;261(6):1681–90. doi: 10.1007/s00417-022-05952-8 (PMC10198863; doi:10.1007/s00417-022-05952-8)
Supplement: Supplementary file 7 — Supplementary file7 (PDF 62.2 KB) [file 417_2022_5952_MOESM7_ESM.pdf]

Table S7

Sensitivity analyses for comparison of aflibercept and ranibizumab

Subgroup – functional and anatomical outcome		Before switch	After switch
**Aflibercept to bevacizumab** (n=32)	**Visual acuity [in LogMAR]**	0.45	0.60
	**CRT [in µm]**	303.8	335.9
	**Macular volume [in mm^3]**	7.9	8.2
**Ranibizumab to bevacizumab** (n=33)	**Visual acuity [in LogMAR]**	0.68	0.76
	**CRT [in µm]**	316.1	336.0
	**Macular volume [in mm^3]**	8.2	8.3

Subgroup – treatment interval		Days
**Aflibercept to bevacizumab** (n=32)	**Before switch**	38.6
	**After switch (initial)[a]**	32.1
	**After switch**	41.8
**Ranibizumab to bevacizumab** (n=33)	**Before switch**	39.4
	**After switch (initial)[a]**	33.5
	**After switch**	37.9

[a]after switch (initial), interval between first and second injection

[b]after switch, interval between penultimate and last injection
